# MRI Segmentation of the Human Brain: Challenges, Methods, and Applications

**DOI:** 10.1155/2015/450341

**Published:** 2015-03-01

**Authors:** Ivana Despotović, Bart Goossens, Wilfried Philips

**Affiliations:** Department of Telecommunications and Information Processing TELIN-IPI-iMinds, Ghent University, St-Pietersnieuwstraat 41, 9000 Ghent, Belgium

## Abstract

Image segmentation is one of the most important tasks in medical image analysis and is often the first and the most critical step in many clinical applications. In brain MRI analysis, image segmentation is commonly used for measuring and visualizing the brain's anatomical structures, for analyzing brain changes, for delineating pathological regions, and for surgical planning and image-guided interventions. In the last few decades, various segmentation techniques of different accuracy and degree of complexity have been developed and reported in the literature. In this paper we review the most popular methods commonly used for brain MRI segmentation. We highlight differences between them and discuss their capabilities, advantages, and limitations. To address the complexity and challenges of the brain MRI segmentation problem, we first introduce the basic concepts of image segmentation. Then, we explain different MRI preprocessing steps including image registration, bias field correction, and removal of nonbrain tissue. Finally, after reviewing different brain MRI segmentation methods, we discuss the validation problem in brain MRI segmentation.

## 1. Introduction

Over the last few decades, the rapid development of noninvasive brain imaging technologies has opened new horizons in analysing and studying the brain anatomy and function. Enormous progress in accessing brain injury and exploring brain anatomy has been made using magnetic resonance imaging (MRI). The advances in brain MR imaging have also provided large amount of data with an increasingly high level of quality. The analysis of these large and complex MRI datasets has become a tedious and complex task for clinicians, who have to manually extract important information. This manual analysis is often time-consuming and prone to errors due to various inter- or intraoperator variability studies. These difficulties in brain MRI data analysis required inventions in computerized methods to improve disease diagnosis and testing. Nowadays, computerized methods for MR image segmentation, registration, and visualization have been extensively used to assist doctors in qualitative diagnosis.

Brain MRI segmentation is an essential task in many clinical applications because it influences the outcome of the entire analysis. This is because different processing steps rely on accurate segmentation of anatomical regions. For example, MRI segmentation is commonly used for measuring and visualizing different brain structures, for delineating lesions, for analysing brain development, and for image-guided interventions and surgical planning. This diversity of image processing applications has led to development of various segmentation techniques of different accuracy and degree of complexity.

In this paper we review the most popular methods commonly used for brain MRI segmentation. We highlight differences between them and discuss their capabilities, advantages, and limitations. To introduce the reader to the complexity of the brain MRI segmentation problem and address its challenges, we first introduce the basic concepts of image segmentation. This includes defining 2D and 3D images, describing an image segmentation problem and image features, and introducing MRI intensity distributions of the brain tissue. Then, we explain different MRI preprocessing steps including image registration, bias field correction, and removal of nonbrain tissue. Finally, after reviewing different brain MRI segmentation methods, we discuss the validation problem in brain MRI segmentation.

## 2. Basic Concepts

### 2.1. 2D and 3D Images

**Figure 1:**
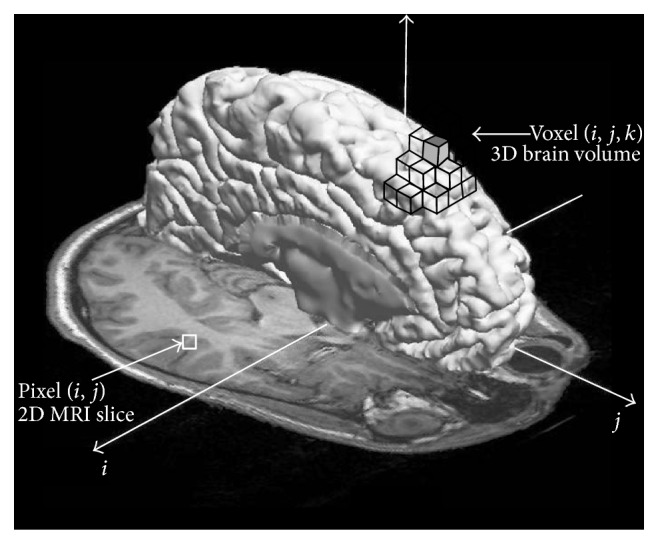
Illustration of image elements in the MRI of the brain. An image pixel (*i*, *j*) is represented with the square in the 2D MRI slice and an image voxel (*x*, *y*, *z*) is represented as the cube in 3D space.

**Figure 2:**
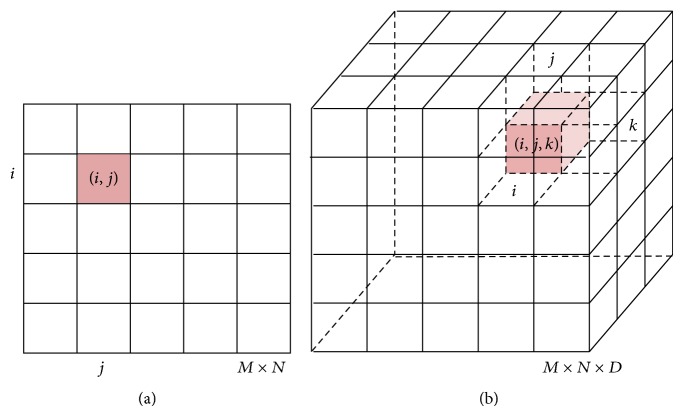
Illustration of image elements in 2D and 3D space. (a) In 2D space image elements (pixels) are represented with lattice nodes depicted as a square. (b) In 3D space image elements (voxels) are represented with lattice nodes depicted as a cube.

An image can be defined as a function *I*(*i*, *j*) in 2D space or *I*(*i*, *j*, *k*) in 3D space, where *i* = 0,…, *M* − 1, *j* = 1,…, *N* − 1, and *k* = 0,…, *D* − 1 denote spatial coordinates. The values (or amplitudes) of the functions *I*(*i*, *j*) and *I*(*i*, *j*, *k*) are intensity values and are typically represented by a gray value {0,…, 255} in MRI of the brain; see Figure 1. Every image consists of a finite set of image elements called pixels in 2D space or voxels in 3D space. Each image element is uniquely specified by its intensity value and its coordinates (*i*, *j*) for pixels and (*i*, *j*, *k*) for voxels, where *i* is the image row number, *j* is the image column number, and *k* is the slice number in a volumetric stack; see Figure 2.

To each image element is assigned a single value based on the average magnetic resonance characteristics present in the tissue corresponding to that element. The size of the element determines the spatial resolution, or the fineness of detail that can be distinguished in an image. Voxel/pixel sizes vary depending on imaging parameters, magnet strength, the time allowed for acquisition, and other factors, but often in standard MRI studies voxel sizes are on the order of 1-2 mm. Greater spatial resolution can be obtained with a longer scanning time, but this must be weighed against patient discomfort. In adult brain MRI studies image acquisition time is around 20 min, while in pediatric MRI studies image acquisition time is limited to between 5 and 15 min.

### 2.2. Image Segmentation

The goal of image segmentation is to divide an image into a set of semantically meaningful, homogeneous, and nonoverlapping regions of similar attributes such as intensity, depth, color, or texture. The segmentation result is either an image of labels identifying each homogeneous region or a set of contours which describe the region boundaries.

**Figure 3:**
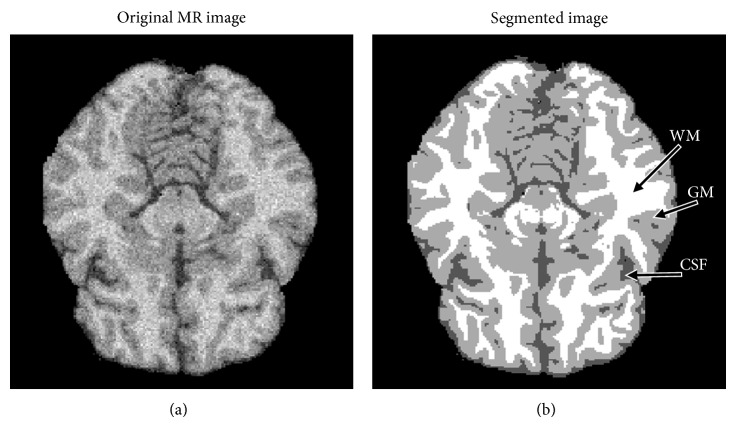
An example of the brain MRI segmentation with an original MR image (a) and segmented image with three labels: WM, GM, and CSF (b).

Fundamental components of structural brain MRI analysis include the classification of MRI data into specific tissue types and the identification and description of specific anatomical structures. Classification means to assign to each element in the image a tissue class, where the classes are defined in advance. The problems of segmentation and classification are interlinked because segmentation implies a classification, while a classifier implicitly segments an image. In the case of brain MRI, image elements are typically classified into three main tissue types: white matter (WM), gray matter (GM), and cerebrospinal fluid (CSF); see Figure 3. The segmentation results are further used in different applications such as for analyzing anatomical structures, for studying pathological regions, for surgical planning, and for visualization.

Image segmentation can be performed on 2D images, sequences of 2D images, or 3D volumetric imagery. Most of the image segmentation research has focused on 2D images. If the data is defined in 3D space (e.g., obtained from a series of MRI images), then typically each image “slice” is segmented individually in a “slice-by-slice” manner. This type of segmenting 3D image volumes often requires a postprocessing step to connect segmented 2D slices into a 3D volume or a continuous surface. Furthermore, the resulting segmentation can contain inconsistencies and nonsmooth surface due to omitting important anatomical information in 3D space. Therefore, the development of 3D segmentation algorithms is desired for more accurate segmentation of volumetric imagery. The main difference between 2D and 3D image segmentation is in the processing elements, pixels/voxels, respectively, and their 2D or 3D neighborhoods (see Section 2.3) over which image features are calculated (see Section 2.4). In practice, 2D image segmentation methods can be extended to 3D space, but often with the cost of an increased complexity of the method and slower computational time.

### 2.3. Modeling the Spatial Context

The use of spatial context or neighborhood information is of great importance in brain MRI segmentation. Unless the image is simply random noise, the intensity of an image pixel/voxel is highly statistically dependent on the gray intensities of its neighbors (surrounding pixels/voxels). Markov random field (MRF) theory provides a basis for modeling local properties of an image, where the global image properties follow the local interactions. MRF models have been successfully integrated in various brain MRI segmentation methods to decrease misclassification errors due to image noise [1–3].

First, let us introduce some notations. As has been described in Section 2.1, every pixel (or voxel) in an image can be represented with one node in the lattice *𝒫*. Let *x*_*i*_ represent an intensity value of a single pixel (or voxel) with a position *i* in an image $\vec{x}=(x_{1},\ldots ,x_{m})$ defined over a finite lattice *𝒫*, where *m* is the total number of image elements (*m* = *MN* for a 2D image and *m* = *MND* for a 3D image). Let *𝒩* = {*𝒩*_*i*_∣∀ *i* ∈ *𝒫*} denote a neighboring system for a lattice *𝒫*, where *𝒩*_*i*_ represent a small neighborhood around *i*, not including *x*_*i*_.

The nodes (pixels/voxels) in a lattice *𝒫* are related to one another via neighborhood system *𝒩* that can be defined as

(1) $$\begin{array}{c} N=\left\{N_{i}\;|\;\forall i\in P\right\}. \end{array}$$

The neighboring relationship has the following properties:

(i) a node *i* does not belong to its own neighborhood: *i* ∉ *𝒩*_*i*_;
(ii) the neighboring relationship is mutual:
  (2) $$\begin{array}{c} i\in N_{i^{\prime }}⟺i^{\prime }\in N_{i}. \end{array}$$

The set of neighbors of *i* can be defined as the set of surrounding nodes within a radius of $\sqrt{r}$ from the center *i*:

(3) $$\begin{array}{c} N_{i}=\left\{i^{\prime }\in P\;|\;{\left[\mathrm{dist}(\text{pixe}{\text{l}}_{i},\text{pixe}{\text{l}}_{i^{\prime }})\right]}^{2}\leq r,i^{\prime }\neq i\right\}, \end{array}$$

where dist⁡(*a*, *b*) is the Euclidean distance between neighboring pixels *a* and *b* and *r* ∈ *ℤ* : *r* ≥ 0 is an integer number.

**Figure 4:**
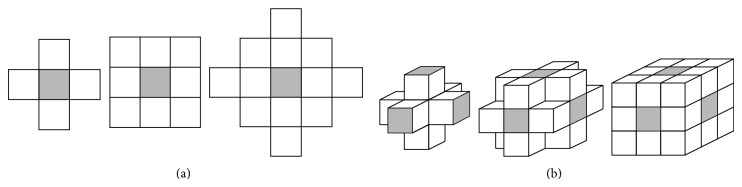
(a) 2D and (b) 3D neighborhood configuration for the first, second, and third order, respectively.

The first and the second order neighborhoods are the most commonly used neighborhoods in image segmentation. The first order neighborhood consists of 4 nearest nodes in a 2D image and 6 nearest nodes in a 3D image, while the second order neighborhood consists of 8 nearest nodes in a 2D image and 18 nearest nodes in a 3D image; see Figure 4.

Markov random field model can be represented with a graph *𝒢*≜(*𝒫*, *𝒩*), where *𝒫* represents the nodes and *𝒩* determines the links (also called edges) that connect the nodes according to the neighborhood relationship. Such graph structure corresponds to an image, where nodes correspond to pixels (or voxels) and the links connecting the nodes represent the contextual dependency between pixels (or voxels). More reading about MRF can be found in [4].

### 2.4. Image Features

Image features represent distinctive characteristics of an object or an image structure to be segmented. Features rely on numerical measurements, including quantitative visual appearance and shape descriptors, that can help to discriminate between the structures of interest and their background. The outcome of image segmentation highly depends on appropriate feature selection (choosing the most relevant features) and accurate feature extraction.

Typically, statistical approach is used for feature extraction and classification in MRI, where pattern/texture is defined by a set of statistically extracted features represented as a vector in multidimensional feature space. The statistical features are based on first and second order statistics of gray level intensities in an image. First order features are derived from the image grey value histogram and include the intensity, mean, median, and standard deviation of the pixel values. Since these features do not incorporate any information on the spatial distribution of the pixel values, they are often used in combination with second order features. Second order descriptors are used to describe image texture and are typically computed using gray level cooccurrence matrix [5]. First and second order features are often called appearance features in the literature. This is because the visual appearance of an object of interest is typically associated with its pixel or voxel intensities (gray values in brain MRI) and spatial interaction between intensities (intensity cooccurrence) in an image.

**Figure 5:**
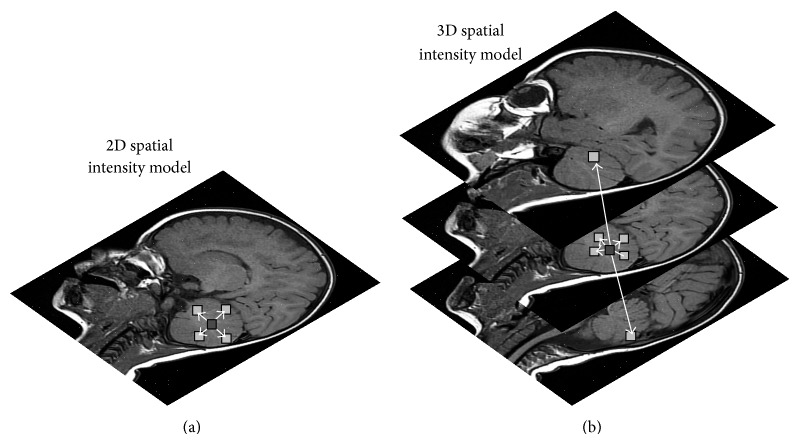
Illustration of 2D (a) and 3D (b) spatial interactions between neighboring pixel/voxel intensities.

Image segmentation based on individual pixel/voxel intensities (first order features) is feasible only when intensities of an object of interest and its background differ to a large extent. Then, the complete object or the majority of its pixels/voxels can be separated from the background by simply comparing the intensity values to the threshold (the intensity value that clearly separates the object from the background). The threshold is derived from the overall intensity distribution of the image. In the presence of image noise and other imaging artifacts, first order features are not sufficient for accurate brain MRI segmentation. In this case more powerful second order discriminative features have to be used that include spatial interaction between intensities. For instance, the appearance of tumour lesion in brain MRI can be associated with spatial patterns of local pixel/voxel intensity variations or empirical probability distributions of intensity cooccurrences. In the spatial interaction models each intensity depends on a subset of the neighboring intensities; see Figures 5 and 4. The most popular models that can capture local spatial interactions between pixels/voxels intensities are MRF models [4].

Additionally, image segmentation performance can be also improved by incorporating probabilistic prior shape models, which have been extensively used in medical image segmentation [6–10]. The probabilistic prior shape models specify an average shape and variation of an object of interest and are typically estimated from a population of coaligned images of the object (training data sets) [11].

One of the most popular features for image segmentation is edges. Edges refer to boundaries of an object surface where the intensities change sharply [12]. Such changes are typically detected by thresholding the first and second order spatial derivatives of the intensities (the intensity gradient and Laplacian). However, edges detected in this way are sensitive to image noise [13] and often require image smoothing as a preprocessing step [14, 15].

Another more robust method for edge detected is the phase congruency method [16, 17], which is a frequency-based method for feature detection. This feature detection method, using local phase and energy, is based on a plausible model of how mammalians detect edges suggested by Morrone and Owens [18] and successfully explains the psychophysical effect of human feature perception. Instead of searching the pixels/voxels in the image with sharp intensity changes, features such as step edges, lines, and corners are detected at points where the Fourier components of the image are maximally in phase.

### 2.5. Intensity Distribution in Brain MRI

**Figure 6:**
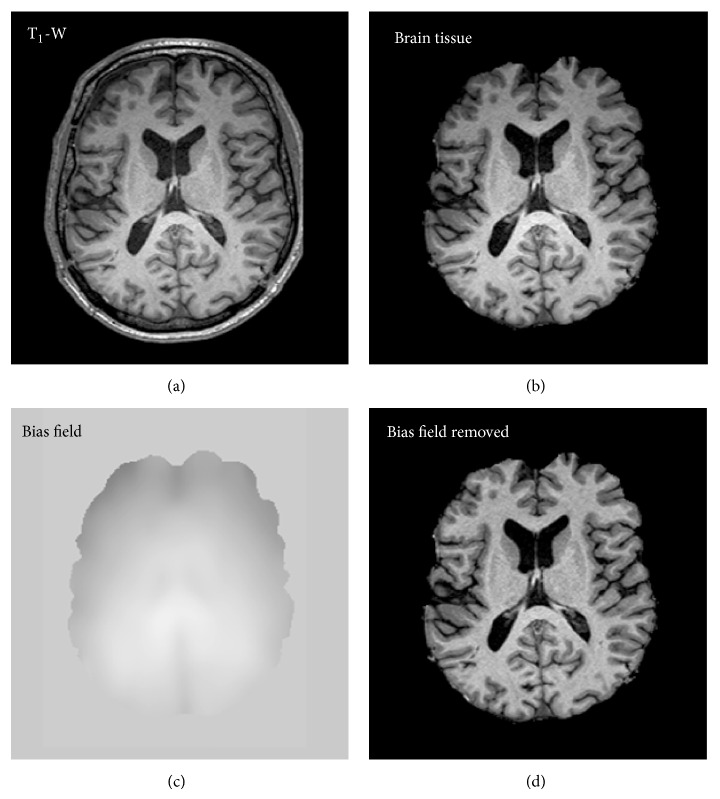
Preprocessing steps: (a) the original T_1_-W MR image of the adult brain; (b) the brain tissue image after removing nonbrain structures; (c) the bias field; (d) the brain tissue image after bias field correction.

The intensity of brain tissue is one of the most important features for brain MRI segmentation. However, when intensity values are corrupted by MRI artifacts such as image noise, partial volume effect (PVE), and bias field effect, intensity-based segmentation algorithms will lead to wrong results. Thus, to obtain relevant and accurate segmentation results, very often several preprocessing steps are necessary to prepare MRI data. For instance, it is necessary to remove background voxels, extract brain tissue, perform image registration for multimodal segmentation, and remove the bias field effect; see Figure 6.

In the case when the bias field, nonbrain structures (e.g., the skull and the scalp) and background voxels are removed, the histogram of the adult brain MRI has three main peaks corresponding to the three main tissue classes; see Figure 8(a). In the healthy adult brain, the intensity variation within tissue is small and the intensities inside the brain can be considered to be a piecewise constant intensity function, corrupted by noise and PVE. The PVE describes the loss of small tissue regions due to the limited resolution of the MRI scanner. It means that one pixel/voxel lies in the interface between two (or more) classes and is a mix of different tissues. This problem is even more critical in imaging of the small neonatal brain. The correction of PVE will be addressed in Section 4.6.

**Figure 7:**
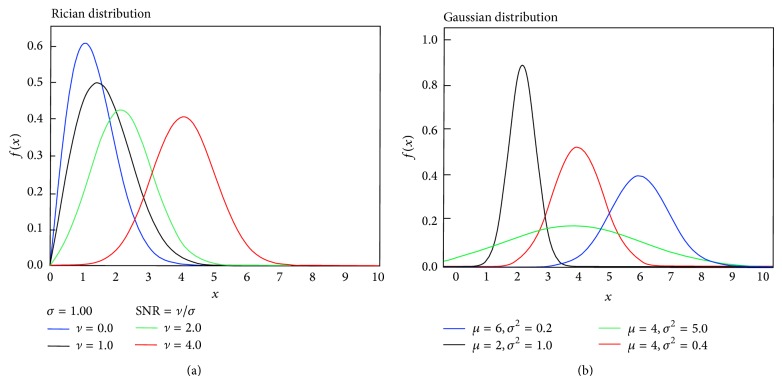
(a) The PDF for the Rician distribution. (b) The PDF for the Gaussian distribution.

It has been shown that the noise in the magnitude images is governed by a Rician distribution, based on the assumption that the noise on the real and imaginary channels is Gaussian [19]. The probability density function for a Rician distribution is defined as

(4) $$\begin{array}{c} f_{\text{Rice}}\left(x\right)=\frac{x}{\sigma^{2}}\exp\left(-\frac{\left(x^{2}+\nu^{2}\right)}{2\sigma^{2}}\right)I_{0}\left(\frac{x\nu }{\sigma^{2}}\right), \end{array}$$

where *x* is the measured pixel/voxel intensity, *ν* is the image pixel/voxel intensity in the absence of noise, *σ* is the standard deviation of the Gaussian noise in the real and the imaginary images, and *I*_0_ is the zero-order modified Bessel function of the first kind. The Rician probability density function (PDF) is plotted in Figure 7(a) for several values of the signal-to-noise ratio (SNR), where the SNR is defined as *ν*/*σ* (the power ratio between the signal and the background noise).

A special case of the Rician distribution is in image regions where only noise is present and SNR = *ν*/*σ* = 0 (e.g., in the dark background areas of an MRI where no NMR signal is present). This special case of the Rician distribution where *ν* = 0 and *I*_0_ = 1 is also known as the Rayleigh distribution:

(5) $$\begin{array}{c} f_{\text{Rayleigh}}\left(x\right)=\frac{x}{\sigma^{2}}\exp-\frac{x^{2}}{2\sigma^{2}}. \end{array}$$

**Figure 8:**
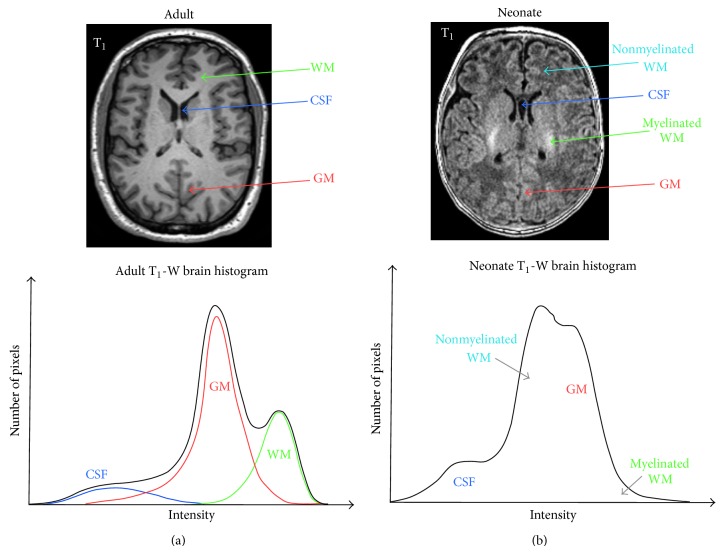
(a) Histogram of a bias-corrected T_1_-W MRI of an adult brain. Histograms of the tissue classes are based on manual segmentation and distributions slightly differ from the Gaussian distribution due to partial volume effect. (b) Histogram of a 1.5 T T_1_-W MRI of a neonatal brain. The difference between the neonatal and the adult brain histogram is the existence of the myelinated and nonmyelinated WM in neonates, which are separated with GM intensities. Since nonmyelinated WM is more dominant than myelinated WM, T_1_-W MRI shows inverted WM/GM intensities in neonates in comparison to adults.

In the image regions where the NMR signal is present and SNR ≥3, the noise distribution approximates a Gaussian distribution; see Figure 7. Thus, the problem of Rician noise in the brain MRI is often simplified in practice by assuming the Gaussian distribution for the noise:

(6) $$\begin{array}{c} f_{\text{Gauss}}\left(x\right)=\frac{1}{\sigma \sqrt{2\pi }}\exp\left(-\frac{{\left(x-\mu \right)}^{2}}{2\sigma^{2}}\right), \end{array}$$

where *x*, *σ*, and *μ* are the intensity, the standard deviation, and the mean value, respectively. Due to this approximation, the histogram of a bias-corrected brain MRI in the presence of noise can be described with a Gaussian mixture model (GMM), where each tissue class (WM, GM, and CSF) is modeled by a Gaussian distribution. However, in the presence of partial volume effects the tissue intensity distributions slightly diverge from a Gaussian distribution, as can be seen from the histogram in Figure 8(a) where histograms of the tissue classes are based on manual segmentation. Recently, the *α*-stable distribution mixture model is also suggested as an alternative to the Gaussian mixture model to model the histogram of MRI data for more complex MRI segmentation [20]. Note that the *α*-stable distribution is a generalization of the Gaussian distribution.

The MRI intensity distribution of the neonatal brain is more complex because the intensity variability within tissue cannot be neglected due to the process of myelination. The histogram of 1.5 T T_1_-W MRI of the neonatal brain is shown in Figure 8(b). The difference between the neonatal and the adult brain histogram is the existence of the myelinated and nonmyelinated WM in neonates, which are separated with GM intensities. Since nonmyelinated WM is more dominant than myelinated WM, T_1_-W MRI shows inverted WM/GM intensities in neonates in comparison to adults.

#### 2.5.1. T_1_-W and T_2_-W Intensity Distribution

It can be noted from the 1D histogram of the bias-corrected T_1_-W MRI of an adult brain in Figure 8(a) that there is an overlap between different tissue classes. Also, it can be seen that an overlap between WM and GM tissue is higher than between GM and CSF. This overlap between the class distributions can cause ambiguities in the decision boundaries when intensity-based segmentation methods are used [21]. However, many researchers showed that adding additional MRI sequences with different contrast properties (e.g., T_2_-W MRI, Proton Density MRI) can improve intensity-based segmentation and help separate the class distributions [22–24]; see Figure 13.

## 3. MRI Preprocessing

After MRI acquisition several preprocessing steps are necessary to prepare MR images for segmentation; see Figure 6. The most important steps include MRI bias field correction, image registration (in the case of multimodal image analysis), and removal of nonbrain tissue (also called a brain extraction).

### 3.1. Bias Field Correction

**Figure 9:**
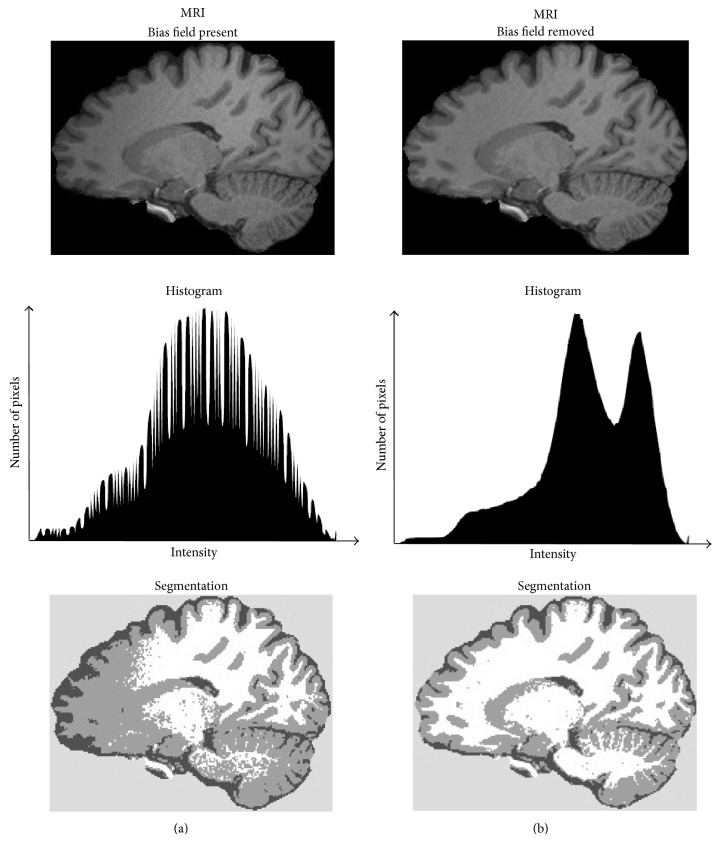
Influence of the bias field on brain MRI segmentation. (a) An example of the sagittal brain MRI slice with bias field is shown in the top of the figure. The image histogram is shown in the middle and the three-label segmentation in the bottom. (b) The bias-corrected MRI slice is shown in the top, the corresponding histogram in the middle, and three-label segmentation in the bottom.

The bias field, also called the intensity inhomogeneity, is a low-frequency spatially varying MRI artifact causing a smooth signal intensity variation within tissue of the same physical properties; see Figure 6. The bias field arises from spatial inhomogeneity of the magnetic field, variations in the sensitivity of the reception coil, and the interaction between the magnetic field and the human body [25, 26]. The bias field is dependent on the strength of the magnetic field. When MR images are scanned at 0.5 T, the bias field is almost invisible and can be neglected. However, when MR images are acquired with modern high-field MR scanners with a magnetic field strength of 1.5 T, 3 T, or higher, the bias field is strong enough to cause problems and considerably affect MRI analysis. In practice, trained medical experts can make visual MRI analysis to certain levels of intensity inhomogeneity (10%–30%) [26]. In contrast, the performance of automatic MRI analysis and intensity-based segmentation methods decreases greatly in the presence of the bias field; see Figure 9. This is because most of the segmentation algorithms assume intensity homogeneity within each class. Therefore, the correction of the bias field is an important step for the efficient segmentation and registration of brain MRI.

The bias field is typically modeled as low-frequency multiplicative field [26, 27]. Suppose that we place all image elements *I*(*i*, *j*, *k*), *i* = 0,…, *M* − 1, *j* = 0,…, *N* − 1, and *k* = 0,…, *D* − 1, into an *m* × 1 column vector $\vec{x}=(x_{1},\ldots ,x_{m})$, where *x*_*i*_, *i* = 1,…, *m*, represents the observed intensity of the *i*th voxel and *m* = *MND* is the total number of image elements. The degradation effect of each image voxel *x*_*i*_ can be expressed as

(7) $$\begin{array}{c} x_{i}=x_{i}^{\prime }b_{i},\;i=1,\ldots ,MND, \end{array}$$

where *x*_*i*_′ is an ideal intensity of the *i*th voxel and *b*_*i*_ is an unknown smoothly varying bias field. The problem of eliminating the bias field is the task of estimating *b*_*i*_.

If the intensities of MRI are logarithmically transformed, the multiplicative bias field becomes an additive bias field as follows:

(8) $$\begin{array}{c} \log\left(x_{i}\right)=\log\left(x_{i}^{\prime }\right)+\log\left(b_{i}\right). \end{array}$$

This simplified multiplicative model is used in most state-of-the-art bias correction methods to represent the bias field [26–28]. However, in reality there are certain limitations to the correctness of this model. Even though the model is consistent with the variations arising from the sensitivity of the receiver coil, the relationship between the measured and true intensities in MRI is more complicated. This is due to nonuniformity of the induced currents and spatial inhomogeneity of the excitation field, which depends on the geometry and electromagnetic properties of the subject as well as the coil polarization and pulse sequence [26]. In spite of these difficulties, the multiplicative low-frequency model is successfully used in practice to model the intensity inhomogeneity in brain MRI.

In the literature, various methods have been proposed to correct the bias field in MRI. One of the earliest methods proposed to correct the bias field is based on the manual labeling of the brain tissue voxels, which are then used to reconstruct the bias field in form of a parametric surface. The main disadvantage of this surface fitting method is the need for manual interaction. The bias field can be also estimated and corrected by using low-pass filtering [29], but this approach can introduce additional artifacts in the image because it also removes the low-frequency component of the true image data. Both the surface fitting method and the low-pass method can be improved and made fully automatic if they are coupled with automatic segmentation of the brain [30, 31]. Other approaches for the bias field correction include minimizing the image entropy [32], fitting the histogram of the local neighbourhood to global histogram of the image [28], maximizing the high-frequency content of the image [26], and using a registered template image [27].

The template is an image/volume which encodes the average probability of finding different kinds of tissues at each spatial location. The anatomical template is obtained by normalizing, aligning, and averaging of anatomical images from several different subjects. All the images are normalized in a standard stereotaxic space such as the Montreal Neurological Institute (MNI space) [33]. MNI is widely used to provide a common reference for the 3D localization of functional activation foci and anatomical structures, enabling the comparison of results obtained across different studies. The standard probabilistic atlas of the human brain consists of a template and three tissue probability maps for WM, GM, and CSF [33]. The tissue probability maps are obtained by normalizing and averaging a number of segmented subjects. The probabilistic atlas then describes the anatomical variability of the brain.

Image registration is a necessary step for the inclusion of probabilistic atlases as a prior knowledge of the brain anatomy into the segmentation method. A probabilistic atlas is often used to initialize and constrain the segmentation process. The prior knowledge of the brain anatomical structures can increase the robustness and accuracy of a segmentation method; see Section 4.3.

### 3.2. Image Registration

Image registration is the process of overlaying (spatially aligning) two or more images of the same content taken at different times, from different viewpoints, and/or by different sensors. Registration is required in medical image analysis for obtaining more complete information about the patient's health when using multimodal images (e.g., MRI, CT, PET, and SPECT) and for treatment verification by comparison of pre- and postintervention images. In medical image registration the term coregistration is used for intrasubject registration (the alignment multimodal images of the same subject), realignment is used for motion correction within the same subject, and normalization is used for intersubject registration when several population groups are studied.

Image registration involves finding the transformation between images so that corresponding image features are spatially aligned. The spatial alignment is typically initialized using rigid or affine transformation [34]. A rigid transformation is a 6-parameter transformation composed of translation and rotation. If scaling and skewing are allowed, we obtain a 12-parameter affine transformation. A rigid registration is sufficient for intrasubject registration if the object of interest does not deform. This is a reasonable assumption for images of the brain if these are acquired at the same stage of brain development. However, if the task is to match images belonging to either different subjects (intersubject registration) or the same subject at different stages of brain development (e.g., growth in children, changes related to ageing, or atrophy due to disease), a nonrigid registration of the images is required to obtain satisfactory results. The nonrigid registration algorithms are typically based on either physical models for transformation such as elastic [35] or fluid deformation models [36] or a linear combination of smooth basis functions [37] or free-form deformations [38]. However, the problems in intersubject brain MRI registration will arise when brains include lesions or diseases, because it is not possible to match the same structures between healthy and diseased brains. A general review of registration techniques can be found in [39–41].

### 3.3. Removal of Nonbrain Tissue

Nonbrain tissues such as fat, skull, or neck have intensities overlapping with intensities of brain tissues. Therefore, the brain has to be extracted before brain segmentation methods can be used. This step classifies voxels as brain or nonbrain. The result can be either a new image with just brain voxels or a binary mask, which has a value of 1 for brain voxels and 0 for the rest of tissues. In general, the brain voxels comprise GM, WM, and CSF of the cerebral cortex and subcortical structures, including the brain stem and cerebellum. The scalp, dura matter, fat, skin, muscles, eyes, and bones are always classified as nonbrain voxels.

**Figure 10:**
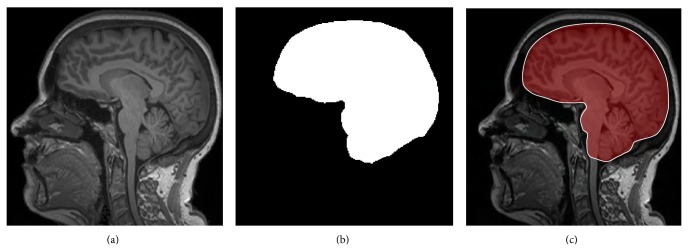
Result of brain extraction on a T_1_ MR image in an axial plane. (a) shows the original T_1_-W MRI. (b) depicts the estimated brain mask. (c) presents an overlap of the brain mask and original MR image.

The common method for brain extraction is to use prior information of the brain anatomy. A deformable template can be registered with an image and nonbrain tissue is then removed by transferring the brain mask from the template [42]. However, brain extraction using a probabilistic atlas is usually not very accurate and can cause misclassification around the brain boundary. An alternative method for extracting the brain is the brain extraction tool (BET) [43, 44], which is part of the publicly available software package FSL. This method finds the center of gravity of the brain and then inflates a sphere until the brain boundary is found. It has been proven to work in practice on good quality T_1_-W and T_2_-W images of the adult brain. An example of the brain extraction is shown in Figure 10.

## 4. MRI Segmentation Methods

In general, MRI segmentation is not a trivial task, because acquired MR images are imperfect and are often corrupted by noise and other image artifacts. The diversity of image processing applications has led to development of various techniques for image segmentation [45–54]. This is because there is no single method that can be suitable for all images, nor are all methods equally good for a particular type of image. For example, some of the methods use only the gray level histogram, while some integrate spatial image information to be robust for noisy environments. Some methods use probabilistic or fuzzy set theoretic approaches, while some additionally integrate prior knowledge (specific image formation model, e.g., MRI brain atlas) to further improve segmentation performance.

However, most of the segmentation methods developed for one class of images can be easily applied/extended to another class of images. For example, the theory of graph cuts, although firstly developed for binary images [55], can be modified and used for MRI segmentation of the brain tissue. Also, unsupervised fuzzy clustering [45, 56, 57] has been successfully applied in different areas such as remote sensing, geology, and medical, biological, and molecular imaging.

The segmentation methods, with application to brain MRI, may be grouped as follows:

- manual segmentation;
- intensity-based methods (including thresholding, region growing, classification, and clustering);
- atlas-based methods;
- surface-based methods (including active contours and surfaces, and multiphase active contours);
- hybrid segmentation methods.

### 4.1. Manual Segmentation

Manual segmentation refers to the process where a human operator (e.g., expert physician) segments and labels an image by hand. This segmentation is typically done in a “slice-by-slice” manner for 3D volumetric imagery. The manual method is believed to be the most accurate because of the difficulty to accurately and reliably delineate structures in medical images. The segmentation difficulties are related to image quality and artifacts.

Given the improvements achieved over the past years by imaging tools (e.g., MR scanners resolve images at millimetric resolution), the manual segmentation has become an intensive and time-consuming task. A trained operator typically has to go through around eighty 512 × 512 images, slice by slice, to extract the contours of the target structures. This manual segmentation is not only tedious but also particularly prone to errors, as assessed by various intra- or interoperator variability studies [58, 59]. Also, manual segmentation results are often difficult and even impossible to reproduce, because even experienced operators show significant variability with respect to their own previous delineation.

However, manual segmentation is still intensively used for defining a surrogate for true delineation (called “ground truth”) and quantitative evaluation of automated segmentation methods. Also, manual segmentation of different brain structures is a fundamental step in brain atlas formation and is used in atlas-based segmentation approaches [50, 51, 60].

For manual delineation, editing tools such as ITK-SNAP [61, 62] usually display 3D data in the form of 3 synchronized 2D orthogonal views (sagittal, coronal, and axial) onto which the operator draws the contour of the target structure. The output data therefore consists of a series of 2D contours from which a continuous 3D surface has to be extracted. This is a nontrivial postprocessing task and is prone to errors. For instance, due to interslice inconsistencies in segmentation, bumps in the reconstructed 3D surface are inevitable. More robust segmentation methods can usually be derived from true 3D structure models in that they can ensure globally smoother and more coherent surfaces across slices.

### 4.2. Intensity-Based Methods

Intensity-based segmentation methods classify individual pixels/voxels based on their intensity. In the case of the brain MRI, three main tissue classes, WM, GM, and CSF, can be distinguished based on intensity; see Figure 3. A more detailed classification is not possible because the intensity profiles of more detailed brain structures overlap. Even separation of the three main tissue classes based on intensity itself requires incorporating tools for dealing with artifacts in MRI, such as intensity inhomogeneity, noise, and partial volume, as well as overlap in intensities of brain and nonbrain tissue (e.g., the scalp has the same intensities as brain tissues).

Several intensity-based techniques are available for tissue classification. The most common method is the use of intensity histogram of all of the voxels and fitting Gaussian functions to the distribution. The probability of a given intensity corresponding to a given type of tissue can thus be inferred and voxels are assigned to tissue types accordingly. Additionally incorporating neighbourhood information helps to give preference to spatially homogeneous regions in the resulting segmentation. This can significantly decrease misclassification due to random noise in the image [1]. Additionally, probabilistic atlases can be included in the classification to inform whether a given location in the brain is likely to contain WM, GM, or CSF voxels [50].

#### 4.2.1. Thresholding

**Figure 11:**
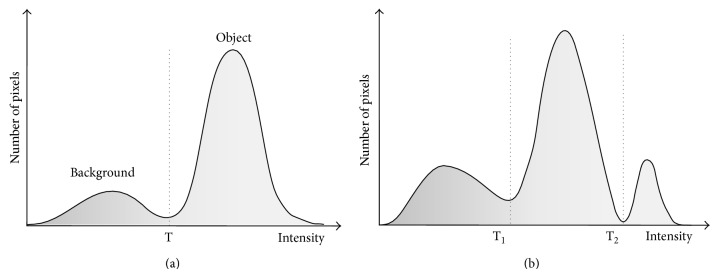
(a) Gray level histogram that can be partitioned by a single threshold. (b) Gray level histogram that can be partitioned by multiple thresholds.

Thresholding is the simplest image segmentation method. A thresholding procedure uses the intensity histogram and attempts to determine intensity values, called thresholds *τ*, which separates the desired classes. The segmentation is then achieved by grouping all pixels between thresholds into one class; see Figure 11. The thresholding methods have many variations: global (single threshold) or local threshold (depending on the position in the image), multithresholding, adaptive thresholding, and so forth. In the case of a single global threshold, segmentation of an image *I*(*i*, *j*) is defined as

(9) $$\begin{array}{c} I^{\prime }\left(i,j\right)=\left\{\begin{array}{cc} 1, & \text{if}\;I\left(i,j\right)>\tau ,\\ 0, & \text{if}\;I\left(i,j\right)\leq \tau , \end{array}\right. \end{array}$$

where *I*′(*i*, *j*) is a segmented (thresholded) image, where pixels labeled with 1 correspond to object and pixels labeled with 0 correspond to background; see Figure 11(a).

Thresholding is fast and computationally efficient method but does not take into account the spatial characteristics of an image (neighborhood information). Thus thresholding is sensitive to noise and intensity inhomogeneities. In low-contrast images it tends to produce scattered groups of pixels rather than connected regions and requires connectivity algorithms as a postprocessing step.

In general, threshold-based segmentation methods are not suitable for textured images. This is because the perceptual qualities of textured images are based on higher order interactions between image elements or objects in the scene. However, in brain MRI segmentation, thresholding can be used to separate background voxels from the brain tissue or to initialize the tissue classes in iterative segmentation methods such as fuzzy *C*-means clustering. A survey on thresholding techniques is provided in [63].

#### 4.2.2. Region Growing

Region growing (also called region merging) is a technique for extracting a connected region of the image which consists of groups of pixels/voxels with similar intensities [64]. In its simplest form, region growing starts with a seed point (pixel/voxel) that belongs to the object of interest. The seed point can be manually selected by an operator or automatically initialised with a seed finding algorithm. Then, region growing examines all neighboring pixels/voxels and if their intensities are similar enough (satisfying a predefined uniformity or homogeneity criterion), they are added to the growing region. This procedure is repeated until no more pixels/voxels can be added to the region.

**Figure 12:**
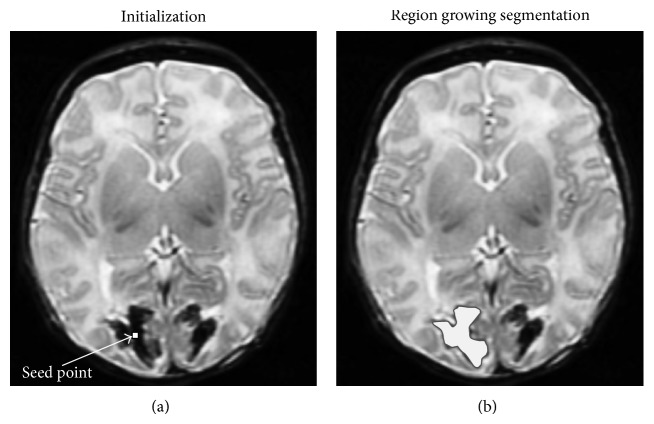
An example of region growing segmentation of a brain lesion. (a) In the initialization step, a seed point is manually selected in the lesion area. (b) The final segmentation result is a connected region and represents the lesion.

Region growing is suitable for segmentation of volumetric images which are composed of large connected homogeneous regions. Thus, it is successfully used in medical image analysis to segment different tissues, organs, or lesions from MR images. For example, it is used in brain MRI analysis for segmentation of brain vessels [65], brain tumour segmentation [66], or extraction of brain surface [67]. See an example of region growing segmentation in Figure 12.

The main disadvantage of the region growing method is its sensitivity to the initialization of seed point. By selecting a different seed point, the segmentation result can be completely different. If seed point and homogeneity criterion are not properly defined, the growing region can leak out and merge with the regions that do not belong to the object of interest. Also, region growing is sensitive to noise and segmented regions in the presence of noise can become disconnected or have holes. On the other hand, separate regions can become connected in the presence of partial volume effects.

#### 4.2.3. Classification Methods

Classification methods use data with known labels to partition image feature space. Image features are typically intensity values but can be also related to texture or other image properties. Classification methods can be both supervised and unsupervised. Supervised classification requires training images, which are manually segmented and then used as references for automatic segmentation of new images. Next to the manual interaction that is laborious and time-consuming, another disadvantage of supervised classification methods is that they generally do not take into account the neighborhood information and thus they are sensitive to noise. Also, the use of the same training set for a large number of images can lead to biased results, which do not take into account anatomical and physiological variability between different subjects.

One of the simplest classifiers is the nearest-neighbor classifier [68], where each pixel/voxel is classified in the same class as the training datum with the closest intensity. A generalization of this approach is the *k*-nearest-neighbor (*k*NN) classifier, where the pixel/voxel is classified according to the majority vote of the closest training data. The *k*NN classifier is considered a nonparametric classifier because it makes no underlying assumption about the statistical structure of the data. It is especially suitable if a large number of training data are available.

The *k*NN classification method was applied to brain MRI segmentation by Warfield et al. [69]. In addition to image intensities, Warfield used spatial localization of brain structures (classes) in form of a nonrigidly registered template as an additional feature to enhance the classification process. The segmentation is then calculated in an iterative process by interleaving the segmentation refinement with updating the nonrigid alignment to the template. This procedure requires manual selection of a large number of training samples for each tissue class to train the *k*NN classifier. Due to the manual interaction in the training phase, the method is not fully automatic and the results depend on particular choice of the training set. Cocosco et al. [70] developed a method for the robust selection of training samples to make the *k*NN classification process fully automatic. This method is reported to deal well with anatomies which differ from the probabilistic atlas. However, it does not deal with the problem of natural intensity variation within each tissue class. Both methods require correction of the bias field as a preprocessing step.

One of the most commonly used parametric classifiers is the Bayesian classifier [30]. The Bayesian classifier models the probabilistic relationships between the attribute set and the class variables, which are then used for estimating the class probability of the unknown variable. This model involves Bayesian inference such as maximum a posteriori (MAP) estimation, where the goal is to estimate the label output image $\hat{x}$ given the observed image **y** by minimizing the* posterior* distribution *P*(**x**∣**y**) of the possible labels **x**:

(10) $$\begin{array}{c} \hat{x}=\arg\underset{\begin{array}{c} x \end{array}}{\max}P\left(x\;|\;y\right). \end{array}$$

The Bayesian framework consists of three probability distributions: the* prior* distribution $P(\vec{x})$, the* posterior* distribution *P*(**x**∣**y**), and the* conditional* distribution *P*(**y**∣**x**) (also called the* likelihood*). The prior distribution embodies the knowledge of likely configurations before an actual image is observed. The posterior distribution is derived after an observation has been made and the likelihood is defined as the probability of obtaining a particular observation given a set of model parameters.

The Bayes rule describes the relation between the posterior probability *P*(**x**∣**y**), prior probability *P*(**x**), and likelihood *P*(**y**∣**x**) as follows:

(11) $$\begin{array}{c} P\left(x\;|\;y\right)=\frac{P\left(y\;|\;x\right)P\left(x\right)}{P\left(y\right)}. \end{array}$$

Using definition (11), the MAP estimate can be written as

(12) x^=argmax⁡xP(y ∣ x)PxPy=argmax⁡x(P(y ∣ x)P(x)),

where *P*(**y**) can be omitted because it is a constant in the case when **y** is known. Since in many cases the probability distributions have exponential functions, this computation can be simplified by using a logarithmic transform:

(13) $$\begin{array}{c} \begin{array}{cc} & \hat{x}=\arg\underset{\begin{array}{c} x \end{array}}{\max}\left(\log P\left(y\;|\;x\right)+\log P\left(x\right)\right). \end{array} \end{array}$$

In the case of the brain MRI segmentation, often it is assumed that the pixel intensities are independent samples from a mixture of Gaussian probability distributions. Training data is collected by obtaining representative samples from each component of the Gaussian mixture accordingly. Classification of new data is obtained by assigning each pixel to the class with the highest posterior probability.

Bayesian classifiers are used in the expectation-maximization (EM) segmentation methods which have been successfully implemented in several software packages used in the medical imaging community: SPM [2], FAST [3], FreeSurfer [21], and 3DSlicer [71]. All these methods implement segmentation and bias correction in the EM framework. They also include various additional improvements, such as nonrigid alignment of atlas [2], including neighbourhood information in the form of Markov random fields [3, 4] or using the *α*-stable distribution mixture model as a generalization of the GMM [20]. More details about the Bayes' theory can be found in [72].

#### 4.2.4. Clustering Methods

Clustering methods are unsupervised segmentation methods that partition an image into clusters of pixels/voxels with similar intensities without using training images. In fact, clustering methods use the available image data to train themselves. The segmentation and training are done in parallel by iterating between two steps: data clustering and estimating the properties of each tissue class. The most commonly used clustering methods are the *k*-means clustering [73], the fuzzy *C*-means clustering [74, 75], and the expectation-maximisation (EM) method [1].

The *k*-means clustering method partitions the input data into *k* classes by iteratively computing a mean intensity for each class (also called centroid) and segmenting the image by classifying each pixel/voxel in the class with the closest centroid. The *k*-means clustering is also known as a hard classification method because it forces each pixel/voxel to belong exclusively to one class in each iteration. The fuzzy *C*-means clustering is soft classification method based on fuzzy set theory [76]. It is a generalization of the *k*-means clustering because it allows each pixel/voxel to belong to multiple classes according to a certain membership value.

The FCM clustering algorithm is based on minimizing the following objective function:

(14) $$\begin{array}{c} J_{m}=\overset{C}{\underset{i=1}{\sum }}\overset{N}{\underset{j=1}{\sum }}u_{ij}^{m}D_{ij}, \end{array}$$

where *N* is the number of image elements that need to be partitioned into *C* clusters, *u*_*ij*_ is the membership function of the element **x**_*j*_ (a feature vector at position *j*) belonging to the *i*th cluster, *m* is the weighting exponent that controls the fuzziness of the resulting partition (most often is set to *m* = 2, if *m* = 1 we have the *k*-means clustering), and *D*_*ij*_ is the similarity measure between **x**_*j*_ and the *i*th cluster center **v**_*i*_. The most commonly used similarity measure is the squared Euclidean distance *D*_*ij*_ = ‖**x**_*j*_ − **v**_*i*_‖^2^.

The objective function *J*_*m*_ (see (14)) is minimized under the following constraints: *u*_*ij*_ ∈ [0,1], ∑_*i*=1_^*C*^ *u*_*ij*_ = 1  ∀ *j*, and 0 < ∑_*j*=1_^*N*^ *u*_*ij*_ < *N*  ∀ *i*. Considering these constraints and calculating the first derivatives of *J*_*m*_ with respect to *u*_*ij*_ and **v**_*i*_ and setting them to zero result in the following two conditions for minimizing *J*_*m*_:

(15) $$\begin{array}{c} u_{ij}={\left[\overset{C}{\underset{k=1}{\sum }}{\left(\frac{D_{ij}}{D_{kj}}\right)}^{1/(m-1)}\right]}^{-1},\\ v_{i}=\frac{\sum_{j=1}^{N}u_{ij}^{m}x_{j}}{\sum_{j=1}^{N}u_{ij}^{m}}. \end{array}$$

The FCM algorithm iteratively optimizes *J*_*m*_, by evaluating (15), until the following stop criterion is satisfied: max_*i*∈[1,*C*]_‖**v**_*i*_^(*l*)^ − **v**_*i*_^(*l* + 1)^‖_*∞*_ < *ϵ*, where *l* is the iteration index and ‖·‖_*∞*_ is the *L*_*∞*_ norm. Once a membership value *u*_*ij*_ for each class *i* is assigned to each pixel *j*, defuzzification of the fuzzy clusters {*F*_*k*_}_*k*=1_^*C*^ into its crisp version {*H*_*k*_}_*k*=1_^*C*^ is done by assigning the pixel to the class with the highest membership value as follows:

(16) $$\begin{array}{c} \underset{i\in \left[1,C\right]}{\max}\left(u_{ij}\right)=u_{kj}⟹x_{j}\in H_{k}. \end{array}$$

The EM method is an iterative method for finding maximum likelihood or MAP estimates of a statistical model. It has the same soft classification principle as FCM method but typically assumes that MRI intensities of different brain tissues can be represented with a Gaussian mixture model. Even though clustering methods do not require training images, they do require some initial parameters and the EM method has shown the highest sensitivity to initialization in comparison to fuzzy *C*-means and *k*-means methods [1].

In general, the EM segmentation framework can be described as follows.

*EM Approach for Brain MRI Segmentation*. Firstly, initialize the EM algorithm. In the case of brain MRI segmentation, the GMM is used to initially estimate model parameters. Then, iterate between expectation step (E-step) and maximization step (M-step) until convergence.

*E*-*Step*. Estimate the brain tissue segmentation given the current estimate of model parameters. This step can include the use of neighbourhood information (e.g., in the form of MRF modeling).

*M*-*Step*. Estimate the model parameters. This step can consist of a combination of the following steps.

1. Estimate the intensity distribution parameters for each tissue class.
2. Estimate the bias correction parameters.
3. Estimate the registration parameters for alignment of probabilistic atlas with the image.

As it is the case with classification methods, clustering methods initially do not incorporate spatial neighborhood information and thus they are sensitive to noise and intensity inhomogeneities. To improve the clustering performance for images corrupted by noise, many extensions of the clustering algorithms have been proposed [49, 57, 77–84]. The most common approach is to include feature information (e.g., intensity values) of the neighboring pixels into the modified FCM objective function [77, 79] or into a similarity measure between cluster centers and image elements [80]. Ahmed et al. [77] modified the objective function of the standard FCM algorithm to allow the immediate neighbours of the pixel to influence its labeling. Chen and Zhang [79] proposed two improvements of the Ahmed et al. algorithm to reduce the computational time. On the other hand, to keep the continuity from the FCM algorithm, Shen et al. [80] introduced a new similarity measure that depends on spatial neighbourhood information, where the degree of the neighbourhood attraction is optimized by a neural network. The clustering performance can also be enhanced by combining pixel-wise fuzzy classification with preprocessing (noise cleaning in the original image) [49, 78] and postprocessing (noise cleaning on the classified data) [78].

**Figure 13:**
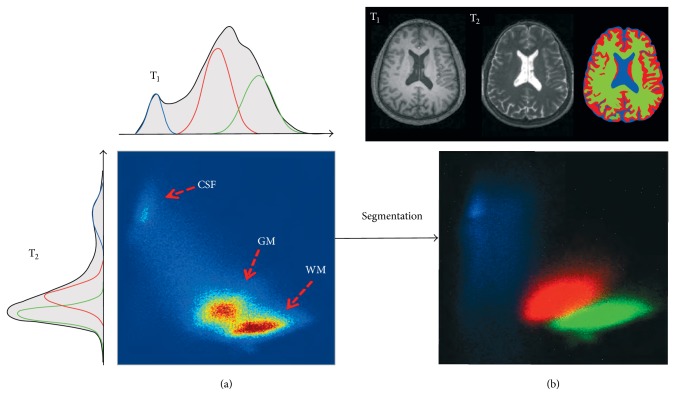
(a) Joint 2D intensity histogram of T_1_-W and T_2_-W MRI of the adult brain. The associated 1D histograms of each MRI modality are plotted on the left and top. Both individual histograms consist of three overlapped Gaussian distributions that approximate the expected tissue distribution of GM, WM, and CSF. (b) The scatter plot of the tissue intensities after applying tissue segmentation. The horizontal axis represents T_1_-W intensities and the vertical axis represents T_2_-W intensities. The red cloud corresponds to GM, the green to WM, and the blue to CSF.

An example of the multimodal T_1_-W and T_2_-W MRI clustering of the adult brain is shown in Figure 13(a). In general, the shape of joints T_1_-W and T_2_-W MRI intensity distributions of different tissue classes depends on the image quality (the presence of noise, PVE, etc.). The shape of the classified data depends on the applied segmentation method. In the example in Figure 13(a), there is a small overlap among classes due to the good quality MRI. Thus, the standard *k*-means clustering method is used to segment the brain tissue probability maps (see Section 4.2.4) and the final clusters are indicated with different colors in the scatter plot of T_1_-W and T_2_-W MRI in Figure 13(b). In general, when MRI artifacts are present and there is a significant overlap among tissue classes, the spatial information of the brain tissue is required to disambiguate the classification problem.

### 4.3. Atlas-Based Methods

If an atlas or template of the human brain for a specific population of interest is available, then atlas-based methods can be a powerful tool for brain MRI segmentation. The atlas contains information about the brain anatomy (e.g., it contains the information about the location of different brain structures) and it is used as a reference (a prior knowledge) for segmenting new images. The main advantage of these methods is the possibility to segment any brain structure available in the atlas without any additional cost. Conceptually, atlas-based approaches are similar to classifier methods, except that they are implemented in the spatial domain rather than in the feature space.

Before a probabilistic atlas can be used as a prior knowledge, it has to be aligned with the image to be segmented. Since the segmentation labels and the “ground truth” are known for the atlas, all atlas information is transferred to the target image after registration. Therefore, the performance of atlas-based methods is directly dependent on quality of the registration method used.

The traditional way of aligning the probabilistic atlas with the image is to use affine registration. Unfortunately, an affine alignment may not be sufficient if the brain anatomy of interest differs significantly from the average atlas anatomy. Pohl et al. therefore suggest aligning the atlas using nonrigid registration [85]. However, in their later work Pohl reports difficulties in registering anatomical template with the image to be segmented using standard registration methods [86]. D'Agostino et al. developed a special similarity measure for registering probabilistic maps directly to the new image [87]. Recently, several methods have been developed which aim to overcome this problem by iteratively refining the segmentation and nonrigid registration of the probabilistic atlas at the same time. Ashburner and Friston developed a method for simultaneous segmentation, bias correction, and nonrigid registration of a probabilistic atlas [2].

However, even with nonrigid registration methods, accurate segmentation of complex structures is difficult due to anatomical variability. Also, atlas-guided segmentation in patients with brain deformations can be difficult and prone to errors, because the probabilistic atlas is based on a population of healthy subjects. For instance, in patients with brain lesions or a brain anatomy that significantly differs from the atlas template, the atlas alignment and the corresponding segmentation of the brain will fail or give inaccurate results. In these cases an atlas-based approach is not a suitable method for image segmentation.

An aligned probabilistic atlas can be also used as a good initial estimate of the segmentation, which is especially important for EM-based methods, as EM algorithm is guaranteed to converge to local, not global, maxima. In addition, most EM-based methods [2, 71] use the probabilistic atlas to constrain the segmentation process where again the correct alignment of the probabilistic atlas is crucial for successful and accurate segmentation.

It is important to note that atlas-based MRI segmentation of the neonatal brain has become a research focus in recent years [42, 51, 53, 88]. MRI segmentation of the neonatal brain tissue is more complex than in adults due to fast growth process, complex anatomy of the developing brain, and often poor MRI quality. Therefore a probabilistic atlas of the newborn brain that contains the spatial variability of the tissue structure is used to segment different brain tissues such as brain cortex, myelinated and nonmyelinated white matter. However, a good atlas of the newborn brain is even more difficult to obtain than in adults, mainly due to the greater anatomical variations between subjects. Therefore, it was necessary to develop a dynamic, probabilistic atlas for any chosen stage of neonatal brain development (for ages of 29 to 44 weeks) [89].

### 4.4. Surface-Based Methods

In addition to intensity-based and atlas-based methods, there are a number of alternative brain MRI segmentation approaches. These approaches include surface-based methods, such as deformable models including active contours and surfaces [46, 52, 54, 61, 90–93].

#### 4.4.1. Active Contours and Surfaces

**Figure 14:**
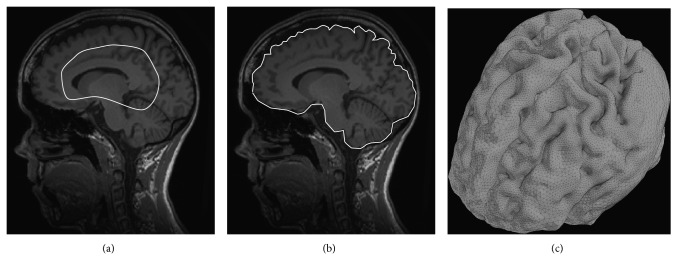
Segmentation of the brain surface using deformable models. (a) A closed curve is initialised inside the brain. (b) The segmentation result of the brain surface in 2D. (c) 3D surface of the brain.

Deformable models are also called active contours or snakes in 2D and active surfaces or active balloons in 3D. Deformable models were introduced by Kass et al. [92] in 2D space and were further developed and generalized in 3D space by Terzopoulos et al. [93, 94]. Deformable models use closed parametric curves or surfaces for delineating region boundaries. The parametric curves and surfaces deform under the influence of external (or image) forces (controlled by the image attributes) and the internal forces, which control the surface regularity. In general, deformable models represent the fusion of geometry, physics, and approximation theory. Geometry is used to represent the shape of the object, physics defines constraints on how the shape may vary over time and space, and approximation theory provides mechanisms for fitting the models to measured data. A visual example of segmentation using deformable models is given in Figure 14.

To delineate a boundary of an object, first a closed curve or surface *𝒮* is placed near the desired boundary in an image. Then, internal and external forces are deforming the curve or surface in an iterative relaxation process where the energy functional is defined as

(17) $$\begin{array}{c} F\left(S\right)=F_{\mathrm{int}}+F_{\text{ext}}. \end{array}$$

The internal forces *F*_int⁡_ are computed from within the curve or surface to keep it smooth throughout the deformation. The external forces *F*_ext_ are usually derived from the image to deform the curve or surface towards the desired feature of interest.

In traditional deformable models, image forces come primarily from the local edge-based information (e.g., based on the gradients of sharp image intensities) [95–97]. However, such reliance on edge information makes deformable models sensitive to noise (e.g., deformable model can leak through noisy edges) and highly dependent on the initial estimate. There have been significant efforts to integrate more global region information into deformable models. The Mumford-Shah model [98] was one of the first region-based methods where the image is approximated using a smooth function inside the regions and not only at their boundaries. Many variants of this model have been proposed later [46, 90]. For instance, Chan and Vese [46] presented a level set method which approximates an image with a constant function inside the regions. Li et al. proposed a region-based level set method for segmentation of MRI in the presence of intensity inhomogeneity. Furthermore, several hybrid deformable models had been later proposed to make use of both local (edge-based) and global (region-based) information [99–102]. Sometimes the image data are not sufficient to delineate the region of interest and thus prior knowledge has to be introduced [103].

#### 4.4.2. Multiphase Active Contours

The most popular Chan-Vese level set method [46] has been successfully used in segmenting images with two distinctive regions (images with binary segmentation energies). In [104], Vese and Chan extended their binary segmentation energies to a multiphase level set formulation. In this way, multiple nonoverlapping regions with spatial consistency and varying characteristics (such as the mean intensities of regions) could be represented with multiple level set functions. This multiphase level set approach was attractive for segmentation of brain MR images which typically have multiple regions of interest with different characteristics. Starting from the Vese and Chan method [104], different extension to multiphase active contours had been developed [54, 105–108]. The advantage of multiphase active contours to other approaches is their robustness to image variations, adaptive energy functionals, topological flexibility, and accurate boundaries.

Traditionally, active contours methods have nonconvex energy minimization due to a gradient descent formulation. In this way, energy minimization converges to undesirable local minima and results in erroneous segmentations. Also, the traditional level set implementation has slower convergence due to discretization errors and the well-known reinitialization requirement. One of the first convex approaches to the two-phase active contours segmentation was proposed by Chan et al. [109]. Later on, several extensions to the more challenging multiphase problem have been proposed [110, 111]. A lot of recent work has been dedicated to developing new multiphase active contours not only with a convex formulation, but also with a reduced computational complexity [54, 105, 107]. Note that globally convex methods are initialization independent.

### 4.5. Hybrid Segmentation Methods

New application-specific brain MRI segmentation problems are emerging and new methods are continuously explored and introduced. Since a selection of the most appropriate technique for a given application is often a difficult task, a combination of several techniques may be necessary to obtain the segmentation goal. Therefore, hybrid or combined segmentation methods have been used extensively in different brain MRI segmentation applications [78, 84, 112–120]. The main idea is to combine different complementary segmentation methods into a hybrid approach to avoid many of the disadvantages of each method alone and improve segmentation accuracy.

Here are some examples of the hybrid brain MRI segmentation methods. Kapur et al. [112] segmented different brain tissues in adults using 2D MRI by combining expectation-maximization segmentation, binary mathematical morphology, and active contours models. Masutani et al. [113] combined model-based region growing with morphological information of local shape to segment cerebral blood vessels. Warfield et al. [120] developed a combined 3D brain MRI segmentation algorithm which iterates between a classification step to identify tissues and an elastic matching step to align a template of normal brain anatomy with the classified tissues. Elastic matching step can generate image segmentation by registering an anatomical atlas to a patient scan.

Furthermore, an unsupervised global-to-local brain MRI segmentation is developed by Xue et al. [78]. They combined minimum error global thresholding and a spatial-feature-based FCM clustering to segment 3D MRI in a “slice-by-slice” manner. In the work of Vijayakumar and Gharpure [117], a hybrid MRI segmentation method, based on artificial neural networks (ANN), is proposed for segmenting tumor lesions, edema, cysts, necrosis, and normal tissue in T2 and FLAIR MRI. More recently, Ortiz et al. [119] suggested an improved brain MRI segmentation method using self-organizing maps (a particular case of ANN) and entropy-gradient clustering.

Hybrid segmentation methods are also used for the neonatal brain segmentation [115, 118]. For example, Despotovic et al. [115] proposed a hybrid strategy to segment the brain volume in neonates using T_1_-W and T_2_-W MRI by combining thresholding, active contours, FCM clustering, and morphological operations. Later on, Gui et al. [118] proposed a morphology-driven automatic segmentation method to segment different anatomical regions of the neonatal brain.

The main drawback of hybrid (combined) segmentation methods is often the increased complexity in comparison with each single method integrated into a hybrid one. This includes a lower computational time and a higher number of different parameters that needs to be tuned for a specific application. Therefore, a hybrid segmentation method should be carefully and wisely designed to give efficient and good quality segmentation.

### 4.6. Partial Volume Effect Correction

As mentioned in Section 2.5, the PVE problem is one of the most common problems in brain MRI segmentation. The PVE describes the loss of small tissue regions because of the limited resolution of the MRI scanner and it is seen on MRI scans as a mix of different tissues in a single pixel/voxel. This effect can cause the misclassification of pixel/voxel that lies in the transition between two (or more) tissues classes. Several methods have been proposed to address the problem of PVE in MRI segmentation for both adult and neonatal brains [3, 42, 103, 121, 122].

One of the first approaches for the partial volume correction is the method by Santago and Gage [123]. They assumed a uniform prior probability for mixed (nonpure) tissues and calculated the intensity distribution of partial volume by minimizing the distance between a model and an image histogram. Another approach is proposed by Nocera and Gee [124], where they used MRF to obtain spatial smooth variations of tissue mixing proportions and a MAP estimation is then computed for partial volume segmentation. Later on, Zhang et al. [3] employ a hidden Markov random field with a finite mixture model to overcome possible PVE and bias field distortions. In the work by Van Leemput et al. [122], a statistical uniform framework for partial volume segmentation is presented without using a heuristic assumption for the prior distribution of mix proportions. They used a parametric statistical image model where each voxel belongs to a single tissue type and introduced an additional downsampling step to cause partial volumes along the borders between tissues. Then, they estimated the tissue mixing proportions by using the expectation-maximization approach.

For all above-mentioned partial volume correction methods, very promising results have been reported for adult T_1_-W MRI. However, these methods rely on the fact that the intensity levels of partial volumes in adult MRI images do not predominantly overlap with the characteristic intensity of any pure tissue class. This assumption is not possible for neonatal MRI due to the inverted graywhite matter contrast and great tissue overlap due to the existence of myelinated WM and nonmyelinated WM; see Figure 8(b). Therefore, several approaches have been proposed to address the PVE in neonatal MRI [42, 103]. Xue et al. [42] proposed method based on expectation-maximization, Markov random field, and atlas information to remove mislabeled voxels and correct errors caused by PVE. They implemented a label propagation strategy to mask off deep GM and myelinated WM, which enabled the segmentation of cortical GM and nonmyelinated WM. In the paper of Wang et al. [103], a rather simple and effective scheme is used to deal with the PVE problem and is based on the anatomical observation that the misclassified WM voxels are surrounded by the CSF and GM and that mislabeled CSF voxels are unconnected from the true WM volume.

## 5. Validation of Brain MRI Segmentation

Validation and quantitative comparison of different segmentation methods are a general problem in medical image analysis. It requires a “ground truth” or gold standard to which the outcome of the segmentation method can be compared. Unfortunately, the “ground truth” does not exist for the analysis of in vivo acquired data in humans. Thus, the “ground truth” of the real patients is typically generated after image acquisition.

In brain MRI analysis, the “ground truth” for the real patient data is usually made by one or more expert physicians who need to manually analyze and segment anatomical structures of interest; see Section 4.1. Although this is the only way to validate the real patient MRI data, this validation must be critically considered because the manual segmentation is prone to errors, highly subjective, and difficult to reproduce (even by the same expert) [59]. Also, this type of validation is not always available because it is time-consuming and depends on the human operator. Therefore, few alternative validation methods evolved in the praxes to validate the accuracy of the segmentation algorithms. The most popular validation methods include the use of software simulations and phantoms.

In software simulations, the artificial MR images are generated with computer programs that simulate the real acquisition process. In this way the “ground truth” is known and the influence of different acquisition parameters and imaging artifacts can be controlled and examined independently. This type of validation is very flexible and easily accessible by different researchers and can be performed with little effort. However, a drawback of this validation is that software simulators cannot take into account all factors that might influence the real image acquisition and the simulated images are only an approximation of the real images.

Since software simulations have certain limitations, validation of new segmentation methods can be done using human-like phantoms, whose physical properties (e.g., geometry of the tissue structures and material properties) are known and are similar to the in vivo properties. The phantom images are generated using the MRI scanner and are more realistic than images generated with software simulations. On the other hand, the phantom images do not offer the flexibility of the software simulations and imaging is more expensive and labour intensive.

The most popular simulated images used for validation of brain MRI segmentation methods are designed by Collins et al. [125] and are also known as a realistic digital brain phantom or simply BrainWeb. Images are freely available online and easily accessible for all researchers to test the performance of the new segmentation methods. The BrainWeb data consists of 181 × 217 × 181 voxel matrix with a resolution of 1 mm ×  1 mm ×  1 mm and is available for different additive noise levels. The noise level (expressed in percentages) is relative to the average real and imaginary values of the overall brightness of the tissue class. The noise is generated using a pseudorandom Gaussian noise, which is added to both real and imaginary components before the final magnitude value of the simulated MR image is computed.

Beside the phantom BrainWeb data, the most popular repository with real MRI data used for validation of brain MRI segmentation methods is the Internet Brain Segmentation Repository (IBSR) [126]. The IBSR repository is also freely available online. It consists of 20 real T_1_-W MRI brain data sets and manually guided expert segmentation results, which are used as a “ground truth” segmentation. Each MRI volume consists of about 60 coronal T_1_-W slices with the interslice resolution of 3.1 mm (thickness between consecutive slices).

To quantify the overlap between the MRI segmentation and the given “ground truth,” several similarity measures are used in the literature. One of the most popular measures used often with BrainWeb data is the Dice coefficient *ρ*_*i*_ [127]:

(18) $$\begin{array}{c} \rho_{i}=\frac{2\left|A_{i}⋂B_{i}\right|}{\left|A_{i}\right|+\left|B_{i}\right|}, \end{array}$$

where *i* stands for a tissue type, *A*_*i*_ and *B*_*i*_ denote the set of pixels labeled into *i* by the “ground truth” and MRI segmentation, respectively, and |*A*_*i*_| denotes the number of elements in *A*_*i*_. The Dice coefficient is in the range 0 ≤ *ρ*_*i*_ ≤ 1 and has value 0 if there is no overlap between the two segmentations and 1 if both segmentations are identical.

The Tanimoto coefficient (also known as the Jaccard index) is often used as a similarity measure with real IBSR data. The Tanimoto coefficient *𝒯*(*i*) for each tissue type *i* is defined as follows:

(19) $$\begin{array}{c} T_{i}=\frac{\left|A_{i}⋂B_{i}\right|}{\left|A_{i}\right|+\left|B_{i}\right|-\left|A_{i}⋂B_{i}\right|}, \end{array}$$

where *A*_*i*_ and *B*_*i*_ denote the set of pixels labeled into *i* by the “ground truth” and the segmentation method, respectively, and |*A*_*i*_| denotes the number of elements in *A*_*i*_. Note that *𝒯*(*i*) ≤ *ρ*(*i*) and 0 ≤ *𝒯*(*i*) ≤ 1.

More readings about similarity measures for evaluation and validation in medical image analysis can be found in [128].

## 6. Discussion and Conclusions

Image segmentation is an important step in many medical applications involving 3D visualization, computer-aided diagnosis, measurements, and registration. This paper has provided a brief introduction to the fundamental concepts of MRI segmentation of the human brain and methods that are commonly used.

In Section 2, we have defined the basic concepts necessary for understanding MRI segmentation methods, such as 2D and 3D image definition, image features, and brain MRI intensity distributions. Following this, preprocessing steps necessary to prepare images for MRI segmentation have been described in Section 3. The most important steps include bias field correction, image registration, and removal of nonbrain tissues or brain extraction. The correction of intensity inhomogeneity is an important step for the efficient segmentation and registration of brain MRI. Image registration is required in brain MRI segmentation for the alignment of multimodal images of the same subject or several population groups taken at different times and from different viewpoints.

Due to the rapid development of medical image modalities, new application-specific segmentation problems are emerging and new methods are continuously explored and introduced. Selection of the most appropriate technique for a given application is a difficult task. In many cases, a combination of several techniques may be necessary to obtain the segmentation goal. Very often integration of multimodal information (acquired from different modalities or over time) can help to segment structures that otherwise could not be detected on single images.

The most popular image segmentation methods that are used for brain MRI segmentation have been reviewed and discussed in Section 4. Newer methods are usually designed to bring more accurate results by incorporating 3D neighborhood information and prior information from atlases. As a consequence, the segmentation process often becomes more complex and time-consuming. The likely future research will still focus not only on developing more accurate and noise-robust methods, but also on improving the computational speed of segmentation methods. Computational efficiency will be particularly important in real-time processing applications such as computer guided surgery.

Probably one of the most important questions concerning medical image segmentation is its use in real clinical settings. It is undeniable that computerized segmentation methods have shown their potentials and applicability in computer-aided diagnosis and therapy planning. It is expected that in the near future they will also become essential tools in real clinical settings, particularly in qualitative diagnosis and where 3D reconstruction and visualization of the anatomical structures are important.
